# Is the Sardinian Blue Zone the New Shangri-La for mental health? Evidence on depressive symptoms and its correlates in late adult life span

**DOI:** 10.1007/s40520-021-02068-7

**Published:** 2022-01-27

**Authors:** Marilena Ruiu, Valeria Carta, Clara Deiana, Maria Chiara Fastame

**Affiliations:** Department of Pedagogy, Psychology, Philosophy, University of Cagliari, Via Is Mirrionis 1, 09123 Cagliari, Italy

**Keywords:** Depression, Aging, Longevity, Blue zone, Physical health, Nutrition

## Abstract

**Background:**

An area of extraordinary longevity (i.e., Sardinian Blue Zone) characterized by a very high prevalence of long-lived successful agers has been validated in Sardinia, an Italian island located in the Mediterranean Sea.

**Aims:**

This study was primarily aimed at examining whether dietary habits (intake of vegetables and fruit, animal-derived proteins, and carbohydrates-rich food), time spent on hobbies, subjective physical health, and socio-cultural context (Sardinian Blue Zone vs. another Sardinian rural area) predicted self-reported depressive symptoms in older adults recruited in the Sardinian Blue Zone and another Sardinian rural area not being characterized by a higher prevalence of long-lived individuals.

**Methods:**

Three hundred and eighteen community-dwellers, age 65 years and older, 188 females and 130 males (*M*_age_ = 79.1 years, SD = 6.9 years) were recruited from the Sardinian Blue Zone and another Sardinian rural area. Each participant individually completed a battery of instruments to assess lifestyle, food habits, perceived physical health, and depressive symptoms through the CES-D inventory.

**Results:**

Significant associations were found between depressive signs, perceived physical health, time spent gardening, proteins, and carbohydrates intake, respectively. Approximately 17% of the variance in the CES-D condition was predicted by socio-cultural context, perceived physical health, and gardening. Participants recruited in the Sardinian Blue Zone spent more time gardening and self-reported better physical health.

**Conclusions:**

current results suggest that a socio-cultural context where people age well (i.e., the Sardinian Blue Zone), and a healthy and physically active lifestyle are crucial for promoting well-being in late adulthood.

## Introduction

One of the direct consequences of the raising in the geriatric population is the need to identify the factors that favor successful aging and that promote the maintenance of the quality of life in later adulthood [1]. Despite the lack of consensus on its definition, successful aging in this context refers to an adaptive process that reflects the interplay between gains and losses in the last decades of life. Specifically, successful aging encompasses compensatory efforts to reduce the detrimental effects of aging through a set of selected adaptive behaviors aimed at empowering residual domains of functioning and dealing with the daily challenges faced by older individuals [2].

There is evidence that subjective mental well-being (e.g., life satisfaction), physical health, social participation, engagement in leisure activities, optimism, and cognitive functioning are crucial correlates of successful aging [3], whereas depression is a persistent menace to it [4]. One potentially relevant approach to evaluate the contribution of psychological characteristics and related factors (e.g., lifestyle) to the enhancement of successful aging is to examine the subjective mental health of long-lived individuals. In this regard, Poulain et al. [5] experimentally validated four rural areas of extraordinary longevity (i.e., Blue Zones) in the world, which are located in Sardinia (Italy), Ikaria (Greece), Okinawa (Japan), and Nicoya (Costa Rica). In short, Blue Zones are limited geographical spots characterized by the highest prevalence of centenarians, where a very traditional lifestyle is conducted and where inhabitants primarily consume local products that usually are cultivated or raised by consumers [6]. Unlike the other Blue Zones, the Sardinian Blue Zone (i.e., SBZ) is a remote mountainous region characterized by the highest quasi gender-balanced concentration of centenarians (i.e., the female/male ratio is approximately 2:1), which is substantially higher than the well-established usual ratio of 5 centenarian females:1 centenarian male [7]. Historically, the inhabitants of the SBZ are mainly shepherds (whereas in the past centuries in the rest of Sardinia the main occupational activity was agriculture), being very keen on preserving their local traditions and conducting a very simple lifestyle [8, 9].

What we are wondering is whether the SBZ is a kind of Shangri-La, a fictional and isolated earthly paradise described by Hilton [10], whose inhabitants get old very slowly, are long-lived, and spend very happy lives characterized by a deep sense of purpose. In this regard, a body of studies revealed that cognitively healthy older people of the SBZ reported fewer depressive symptoms and greater psychological well-being than peers living in the Sardinian town of Sassari and a rural area of northern Italy [11]. Extending this, recently Fastame et al. [12] found that older people of the SBZ reported better life satisfaction and optimism than a group of peers living in the Sardinian capital (i.e., Cagliari metropolitan area).

A further stream of research documented that a regular and vigorous physical activity assessed in terms of time spent in aerobic exercise, gardening, or walking [13, 14], and the participation in leisure activities promoting social engagement and mental stimulation might be significant determinants of successful aging [4, 15]. In this regard, there is evidence that older adults of the SBZ displaying an active lifestyle (i.e., they were primarily engaged in outdoor socio-cultural leisure activities and gardening) reported better psychological well-being than sedentary participants residing in the same area [16]. In addition, Fastame et al. [17] conducted the first follow-up study in a Blue Zone to examine the developmental trajectories associated with the motor and psychological profile in late adulthood. The authors documented that, compared to the national normative values, older individuals of the SBZ who were physically active (i.e., they spent many hours gardening) and engaged in outdoor leisure activities, reported greater psychological well-being, whereas depressive symptoms were stable over 2 years. According to Pes et al. [9, 18] the high levels of physical activity (i.e., due to the slope of the territory that makes more intense the cardiovascular activity of the inhabitants), significant consumption of fruits and vegetables, cereal-derived foods, and moderate intake of local dairy products promote the longevity among the residents of the SBZ. In line with this, additional investigations conducted elsewhere revealed that greater psychological well-being is associated with higher fruit and vegetable consumption in late adulthood [e.g., 19. Moreover, a study carried out in the countries located in the Mediterranean Sea (e.g., Cyprus, Greece) showed that older participants that regularly use the Mediterranean diet (i.e., characterized by a high intake of plant food, olive oil and cheese, and low-to-moderate consumption of eggs, red meat, and fish) reported fewer depressive symptoms [20]. Extending this, according to Gopinath et al. [21], higher vegetables and fruits consumption is associated with a lower likelihood of depressive symptoms in older adults, whereas no significant associations have been found between the intake of carbohydrates and self-reported depressive signs assessed through the Center for Epidemiological Studies-Depression Scale [22] (see the Materials section).

But if a tradition of research is corroborating the idea that older people of the SBZ are physically healthy and self-report better psychological well-being than peers recruited in Sardinian urban areas, or rural villages located in northern Italy [11, 12], very little is known about the impact of lifestyle on the perceived mental health of older people living in Sardinian rural spots not belonging to the longevity area. Concerning this issue, Fastame et al. [23] recently documented that gardening was a significant predictor of depressive symptoms [22] self-assessed by a sample of older participants enrolled in the village of Arborea. Furthermore, there is evidence that time spent in leisure activities positively correlated with hedonic (i.e., feeling satisfied with life, well and happy) and eudaimonic (i.e., having a life full of purpose, meaning, and values) well-being, and fluid intelligence of older Sardinians living in other rural areas [24].

To date, no studies have concurrently explored the nature of the associations between perceived physical health, lifestyle, food habits, and self-reported depressive symptoms in the Blue Zones. In our opinion addressing this issue is crucial for the impact that the aforementioned factors might have in terms of successful aging. Therefore, the current investigation intended to overcome this gap, that is, the primary goal was examining whether dietary habits, perceived physical health, socio-cultural context (i.e., living in the SBZ vs. living in another Sardinian rural area), time spent gardening, and outdoor leisure activities predicted depressive signs in late adulthood. A further goal was to explore the effect of socio-cultural context on perceived physical health, and lifestyle (i.e., time spent gardening and leisure activities) when the effect of age was controlled for. Finally, the effect of the socio-cultural context on the age at death of participants’ parents was examined.

Following Boehm et al. [19] and Gopinath et al. [21], it was hypothesized that higher vegetables and fruit consumption was associated with the occurrence of fewer depressive symptoms. In addition, it was expected that the intake of carbohydrate-rich food was not associated with the occurrence of self-reported depressive signs [21]. Moreover, physical health and engagement in gardening and leisure activities were expected to predict perceived negative mood [9, 16, 23]. In addition, extending previous evidence [12] residential context (i.e., SBZ vs. Sardinian rural area) was expected to be a significant predictor of perceived negative mood, that is, older residents of the SBZ were expected to self-report fewer depressive symptoms than participants enrolled in another Sardinian area. Finally, a priori further hypotheses were not proposed, since relevant evidence on the impact of socio-cultural context on the lifestyle of older adults living in the SBZ and in other Sardinian rural villages lacks.

## Methods

### Participants

This study was conducted in several experimentally validated villages in the SBZ and in another Sardinian rural area that is not known for the extreme longevity of its inhabitants. The recruitment of the participants was carried out through personal contacts with local social agencies and by word-of-mouth into the community.

Three hundred and eighteen 65–99-year-old community-dwellers, 188 females and 130 males (*M*_age_ = 79.1 years, SD = 6.9 years) were recruited from the SBZ (*n* = 150, *n* females = 92) and in other Sardinian rural villages (*n* = 168, *n* females = 96), and took voluntarily part in the study. In line with Fastame et al. [16, 17], the following inclusion criteria had to be satisfied to participate in the study: (1) to be born and resident in the area in which the participant was recruited; (2) to be a community-dweller; (3) to be a descendant of families living in that area for at least two previous generations; (4) to display a score > 20 in the Mini-Mental State Examination (MMSE) [25], indicating the lack of severe signs of cognitive decline; 5) living in the SBZ or in another Sardinian rural area not being characterized by extreme longevity, as reported by Poulain et al. [7].

Area of residency (i.e., SBZ vs. other Sardinian rural village) was equally distributed across the participants (*χ*^2^ = 1.019, *df* = 1, *p* = 0.313). Moreover, gender (*χ*^2^ = 0.576, *df* = 1, *p* = 0.448) and marital status (*χ*^2^ = 2.801, *df* = 1, *p* = 0.094) were equally distributed across the areas of residency (i.e., SBZ vs. No Blue Zone). In contrast, being regularly engaged in gardening (i.e., yes vs. no, *χ*^2^ = 20.190, *df* = 1, *p* = 0.001) or outdoor leisure activities (i.e., yes vs. no, *χ*^2^ = 11.815, *df* = 1, *p* < 0.001) were not counterbalanced. Indeed, SBZ respondents spent their time both gardening (*n* = 95/150) and performing outdoor leisure activities (*n* = 107/150), whereas participants living in the other rural area reported to do less physical activity (*n* ‘yes gardening’ = 64/168) but were primarily engaged in outdoor recreation (*n* = 146/168).

### Materials

The 20-item MMSE [25] was used to screen the global cognitive function of the respondents. Indeed, this tool assesses different cognitive processes, such as working memory, attention, long-term memory, spatial orientation. Following Magni et al. [26], scores were adjusted for years of formal education and age (maximum score = 30). A score < 20 indicates the occurrence of suspected severe signs of cognitive decline.

A socio-demographic interview was proposed to collect some information on socio-demographic characteristics (e.g., age, gender, marital status, years of education), lifestyle (e.g., gardening: yes vs. no, time per week spent to perform outdoor socio-recreational activities, time per week spent gardening), and dietary habits (i.e., percentage of fruit and vegetables intake calculated per week, percentage of animal-derived proteins consumed per week, percentage of carbohydrate-rich food consumed per week) of our respondents.

The Center for Epidemiological Studies-Depression Scale (CES-D) [22, 27] encompasses 20 items that assess the occurrence of depressive symptoms during the previous week. For each item, the respondent had to self-rate his/her level of negative mood on a four-point Likert scale ranging from 0 (Rarely or Never) to 3 (Most days or Every day). The total score ranges from 0 to 60. A score ≥ 16 indicates the presence of significant depressive signs, whereas a score ≥ 23 is the Italian cut-off used to detect suspected cases of major depression.

The Perceived Physical Health index [28] is composed of a single item that assesses the subjective level of physical health on a Likert scale ranging from 0 (worst health) to 10 (excellent health).

### Procedure

The study was conducted in accordance with the 1964 Declaration of Helsinki and its later amendments. The study is consistent with the ethical standards of the institutional research committee that approved it. Written informed consent was given by all participants before participation.

Each participant was individually tested in a quiet room in his/her home. First, the MMSE test was presented, and if severe signs of cognitive deterioration (score < 20) were excluded, the socio-demographic interview was presented. Then the order of administration of the additional tools was counterbalanced across the participants. To avoid the fatigue effect of the participants, the examiners read aloud each statement and recorded the responses given by the participants. Each session lasted approximately 40 min.

### Statistical analyses

Following Brysbaert [29], an a priori power analysis using the G-power program [30] indicated that to perform two-tailed correlational analyses a convenient sample of 255 people would be needed, when *r* = 0.2, power = 0.9, with alpha at 0.05. Moreover, to conduct regression analysis using 8 predictors, with 90% power, alpha at 0.05, and moderate effect size (*f*^2^ = 0.15), an a priori power analysis established that 136 participants would be needed. Finally, to conduct an Analysis of Covariance (ANCOVA) with two groups and one covariate, with 90% power, alpha at 0.05, and expecting moderate effect size (*f* = 0.25), at least 171 participants would be required.

Data were analyzed using IBM SPSS Statistics version 24 (SPSS Inc., Chicago, IL, USA). Descriptive statistical analyses were conducted to illustrate the characteristics of the sample. Pearson’s correlations were computed to examine the nature of the relationships between CES-D, age, amount of food intake, subjective physical health, and hours spent on leisure activities and gardening and to check for multicollinearity. Then, based on the results of the correlational analyses, a hierarchical multiple regression analysis was carried out to explore what factors predict self-reported depressive symptoms. Furthermore, three ANCOVAs were carried out to examine the impact of socio-cultural context on perceived physical health, time spent gardening, and time spent in leisure activities, while the effect of age was controlled for. Finally, two Analyses of Variance (ANOVAs) were conducted to explore the socio-cultural context effect on the age of death of participants’ parents. The 0.05 level of significance was adopted throughout all analyses.

## Results

The descriptive analyses revealed that our sample did not show evident signs of cognitive impairment, since the mean MMSE score of the sample was 26.6 (SD = 2) and according to the Italian norms, a score ≥ 24 is considered an index of normal cognitive function. Moreover, compared to the Italian cut-off, the mean CES-D score of the participants recruited in the SBZ and the further Sardinian rural areas was far from the critical value (i.e., ≥ 23) indicating the suspected occurrence of clinical depression. Indeed, participants living in the SBZ (*M* = 8.41, SD = 6.4) and those recruited in other Sardinian rural villages (*M* = 13.55, SD = 8.2) reported few depressive signs, that is, the mean CES-D score was even below the cut-off (i.e., ≥ 16) indicating the occurrence of significant depressive symptoms. However, a data inspection revealed that 4 females recruited in the SBZ and 17 females and 7 males residing in other Sardinian rural villages reported a CES-D score ≥ 23, indicating the suspect occurrence of clinical depression.

Then, Pearson’s product-moment correlations were calculated between CES-D, age, perceived physical health, weekly time spent in leisure activities and gardening, percentage of vegetables and fruits intake, percentage of carbohydrate-rich foods, and percentage of animal-derived proteins intake. Following Pallant [31] the variables examined were not highly correlated with each other (*r* ≥ 0.9); therefore, no evidence of multicollinearity between them was found. Table 1 summarizes these findings.

**Table 1:** Zero-order correlations between self-reported depressive symptoms (i.e., CES-D), age, perceived physical health (i.e., PHYS), intake of carbohydrates (i.e., Carbohydrates), intake of animal-derived proteins (i.e., Proteins), intake of fruit and vegetables (i.e., Vegetables), time spent gardening (i.e., Gardening), time spent in leisure activities (Hobbies), respectively

	1	2	3	4	5	6	7	8
1. CES-D	–							
2. Age	0.105	*–*						
3. PHYS	− 0.204**	− 0.067	–					
4. Carbohydrates	− 0.112*	0.043	0.140*	–				
5. Proteins	− 0.139*	− 0.137*	0.142*	0.279**	–			
6. Vegetables	− 0.095	− 0.076	0.131*	0.162***	− 0.046	–		
7. Gardening	− 0.193**	− 0.182*	0.049	0.099	0.145**	− 0.049	–	
8. Hobbies	0.088	− 0.158**	0.073	− 0.147**	− 0.041	− 0.024	− 0.067	–

*p < 0.05

**p < 0.01

***p < 0.001

Moreover, based on the results of the correlational analyses, a hierarchical multiple regression analysis was conducted. Residential context (i.e., 0 = SBZ vs. 1 = another Sardinian rural area) was entered at Step 1, physical health index was entered at Step 2, carbohydrates and proteins intake scores were inserted at Step 3, and time spent gardening was inserted at Step 4, whereas the CES-D score was entered as the dependent variable. Preliminary analyses were conducted to ensure no violation of the assumptions of normality, linearity, multicollinearity, and homoscedasticity (tolerance value < 0.10, or a VIF value > 10). After entry of the time spent gardening at Step 4, the total variance explained by the model as a whole was approximately 17% [*F* (5,311) = 12.464, *p* < 0.001]. Table 2 illustrates these results.

**Table 2:** Summary of a hierarchical regression analysis for depressive signs assessed through the CES-D questionnaire

	*B*		95% CI for *B*	*SE B*	*β*	*R*^2^	Δ*R*^2^
Variable		*LL*	*UL*				
Step 1						0.10	0.10***
Constant	8.413***	7.233	9.593	0.60			
Residential context	5.012***	3.386	6.638	0.826	0.329***		
Step 2						0.13	0.03***
Constant	14.448***	10.584	18.312	1.964			
Residential context	4.660***	3.044	6.277	0.822	0.30***		
PHYS	− 0.798**	− 1.285	− 0.311	0.248	− 0.17**		
Step 3						0.15	0.02***
Constant	15.576***	10.650	20.502	2.504			
Residential context	4.789***	3.114	6.464	0.851	0.31***		
PHYS	− 0.726**	− 1.217	− 0.236	0.249	− 0.16**		
Carbohydrates	0.121	− 0.384	0.629	0.257	0.03		
Proteins	− 0.554*	− 1.029	− 0.080	0.241	− 0.13*		
Step 4						0.17	0.02***
Constant	15.867***	10.984	20.750	2.482			
Residential context	4.581***	2.915	6.247	0.847	0.30***		
PHYS	− 0.718**	− 1.204	− 0.232	0.247	− 0.15**		
Carbohydrates	0.142	− 0.358	0.642	0.254	0.03		
Proteins	− 0.472	− 0.946	0.002	0.241	− 0.11		
Gardening	− 0.088**	− 0.152	− 0.023	0.033	− 0.14**		

Residential context, 0 = Sardinian Blue Zone, 1 = other Sardinian rural area; PHYS = perceived physical health; Carbohydrates = percentage of food rich in carbohydrates weekly consumed; Proteins = percentage of animal-derived food weekly consumed; Gardening = amount of time per week spent gardening

*CI* confidence interval, *LL* lower limit, *UL* upper limit

*p < 0.05

**p < 0.01

***p < 0.001

In addition, three separate ANCOVAs were performed to explore the effect of area of residency (i.e., SBZ vs. another Sardinian rural area) on perceived physical health, time spent gardening, and time spent in outdoor leisure activities, while age was used as a covariate. The main effect of residential area was significant in physical health [*F*(1,315) = 6.348, *p* = 0.012, *ηp*^2^ = 0.020], time spent gardening [*F*(1,315) = 5.546, *p* = 0.019, *ηp*^2^ = 0.017], and time spent in leisure activities [*F*(1,315) = 41.425, *p* < 0.0001, *ηp*^2^ = 0.12] conditions, respectively. The effect of the covariate was significant only in the time spent gardening [*F*(1,315) = 12.642, *p* < 0.0001, *ηp*^2^ = 0.039] and time spent in leisure activities conditions [*F*(1,315) = 5.060, *p* = 0.025, *ηp*^2^ = 0.016], whereas the effect of age was not significant in the perceived physical health condition [*F*(1,315) = 2.230, *p* = 0.136]. Older adults living in the SBZ spent more time performing physical activity in the garden (*M* = 10 h per week, SD = 11.7) than peers living in other rural villages (*M* = 7.3 h per week, SD = 13). In contrast, the latter group spent more time carrying out leisure activities (*M* = 16 h per week, SD = 12.6) than older individuals of the SBZ (*M* = 7.8 h per week, SD = 8.5). Moreover, participants recruited in the SBZ self-reported greater physical health (*M* = 7.57, SD = 1.49) than the group living in another Sardinian rural area (*M* = 7.13, SD = 1.77).

Finally, two ANOVAs documented the significant main effect of area of residency on the age at death of participants’ fathers [*F*(1,316) = 9.153, *p* = 0.003, *ηp*^2^ = 0.028] but not on the age at death of participants’ mother [*F*(1,316) = 1.621, *p* = 0.204]. The mean age at death of participants’ fathers recruited in the SBZ was 78.6 years (SD = 12.4), whereas the mean age at death of participants’ fathers recruited in another Sardinian rural area was 74.2 (SD = 13.5). The mean age at death of participants’ mothers enrolled in the SBZ was 82.2 years (SD = 12.7), whereas the mean age at death of participants’ mothers living in another Sardinian rural area was 80.1 (SD = 15.2).

## Discussion

The primary goal of this study was to examine the role played by residential context, perceived physical health, lifestyle (i.e., time spent in leisure activities and gardening), and dietary habits as predictors of self-reported depressive symptoms in a sample of older individuals living in the SBZ and an additional Sardinian rural area. Overall, the current findings extend previous evidence, suggesting that the SBZ is an area where its older inhabitants self-report better perceived mental health. Indeed, in line with Fastame et al. [11, 16], the data inspection revealed that participants with suspected major depression (i.e., CES-D score ≥ 23) were primarily females who did not live in the area of extraordinary longevity but resided in another Sardinian rural area.

Moreover, the current findings documented that unlike our initial hypotheses based on previous findings [19, 21], the intake of fruit and vegetables did not have any beneficial influence on the occurrence of self-reported depressive signs. Furthermore, unlike Gopinath et al. [21], we found that the consumption of carbohydrate-rich food was negatively associated with the CES-D score. In addition, it was also documented that those who were more engaged in active physical activity reported a greater animal-derived protein intake, which, in turn, was negatively associated with depressive symptoms. That is older participants reporting fewer negative emotions also referred to assume more foods like pasta, cookies, and meat and spent more time gardening. On the one hand, these outcomes are consistent with previous evidence [32] documenting that the combination of moderate to vigorous physical activity and adequate protein intake is crucial to maintain physical strength and to prevent sarcopenia (i.e., a condition characterized by the loss of muscle mass) in late adulthood. On the other hand, following Wansink et al. [33], it is plausible to hypothesize that our participants used carbohydrate-rich food and animal-derived proteins as comfort food, that is, as a strategy to alleviate negative emotions.

Extending previous evidence [9, 16], small but significant negative correlations [34] were also found between the CES-D score, time spent gardening, and perceived physical health, respectively. That is, in line with previous studies [e.g., 13, 14, 23, older individuals more engaged in vigorous and regular physical activity reported fewer depressive symptoms and had a more positive image of their functional status.

Furthermore, the regression analysis revealed that a modest but significant percentage of variance in the CES-D condition was explained by the socio-cultural context of residence, perceived physical health, and time spent gardening. In addition, three ANCOVAs documented the impact of socio-cultural context on the perceived physical health and lifestyle of our participants. Altogether, we found that participants recruited in the SBZ exhibited fewer depressive symptoms, reported better physical health, and spent more time gardening than older individuals recruited in the other Sardinian rural area, which, in turn, were more engaged in other leisure activities. Far from assuming that the actual Shangri-La is located in Sardinia, extending previous research [3, 12, 17, 18], this study documented the significant impact of the socio-cultural environment, time regularly engaged in physical activity, and perceived functional health to promote subjective mental health in late adulthood. Specifically, consistently with previous studies [8, 12], the significant contribution of the socio-cultural context as a predictor of depressive signs (i.e., 10% of the variance relative to the CES-D score) let us assume that living in a socio-cultural context such as the SBZ, in which older individuals are autonomous in facing their daily life, play an active role in their community, and are considered a resource for passing on local traditions and cultural values to younger people contributes massively to the maintenance of high levels of mental health in late adulthood [11, 12]. Moreover, as documented elsewhere [e.g., 15, 28, regular physical activity and perceived functional health impact subjective mental health. Finally, we also found that the fathers of our participants enrolled in the SBZ lived longer than the fathers of the participants recruited in another Sardinian area. Overall, these findings extend those documented by Pes et al. [9], about the beneficial effects of physical activity and nutritional habits on male longevity in the SBZ.

Embracing an applied perspective, these outcomes suggest that maintaining an active and healthy lifestyle promotes the mental and physical well-being of older individuals. Moreover, extending previous evidence [35], the current findings suggest that the implementation of specific interventions involving older adults where they can feel competent in performing certain activities (e.g., sport) may be effective in boosting self-efficacy and mental well-being in late adulthood. Consistent with this, there is evidence that motor losses negatively impact older individuals’ ability to manage activities of daily living (e.g., self-care, shopping), causing a significant reduction in their social participation and autonomy, which, in turn, could affect their mental health [36]. Therefore, the implementation of specific interventions aimed at promoting physical activity (e.g., regular aerobic exercise) and cognitive functioning of older individuals should be encouraged to enhance their functional and mental well-being and might be useful to maintain independence in daily living and prevent cognitive decline [15].

However, this study has some limitations, such as the exclusive recruitment of community-dwellers, paucity of tools examining perceived mental health, lack of longitudinal evidence, and lack of an exhaustive clinical assessment to diagnose or exclude major depression cases. Concerning the last critical point, a recent study conducted on the adult population showed that suicide mortality is very high in the rural municipalities located in the southern-eastern part of Sardinia (i.e., a territory including also the SBZ) [37]. In line with this evidence, another investigation conducted in Sardinian urban and rural areas documented that suicidal acts (i.e., attempts or actual suicides) were lower in the metropolitan area of Cagliari than in the remaining Sardinian territory [38].

According to the authors [37, 38], social isolation (favored by the village altitude and lower population density), occupational difficulties, difficult access to public services (e.g., schools, banks, pharmacies), family history of psychiatric disorders, abuse of substances (including alcohol), and low per capita income are risk factors for the suicide of Sardinian adults. However, controversial evidence has been found concerning the gender effect—one study documented higher suicide mortality among men [37], whereas the other one revealed that suicidality (i.e., ideation or act) is more likely among females [38]. Furthermore, no specific information has been documented about suicide mortality or suicidality ideation among Sardinian older adults. Therefore, future research is needed to overcome the above-mentioned issues and to replicate this study in further rural areas characterized by extraordinary longevity, such as Okinawa, where low levels of depression and close family and non-family relationships have been reported as in the SBZ [6, 39].

## Data Availability

The data that support the findings of this study are not publicly available due to privacy or ethical restrictions.
